# An 8-Week Program of Plyometrics and Sprints with Changes of Direction Improved Anaerobic Fitness in Young Male Soccer Players

**DOI:** 10.3390/ijerph181910446

**Published:** 2021-10-04

**Authors:** Ghaith Aloui, Souhail Hermassi, Aymen Khemiri, Thomas Bartels, Lawrence D. Hayes, El Ghali Bouhafs, Mohamed Souhaiel Chelly, René Schwesig

**Affiliations:** 1 Research Unit (UR17JS01) Sport Performance, Health & Society, Higher Institute of Sport and Physical Education, University of La Manouba, Ksar-Saîd, Tunis 2010, Tunisia; gaithaloui@hotmail.fr (G.A.); aymenkha3@gmail.com (A.K.); mohamedsouhaiel.chelly@issep.uma.tn (M.S.C.); 2 Physical Education Department, College of Education, Qatar University, Doha 2713, Qatar; 3 Center of Joint Surgery, Sports Clinic Halle, 06108 Halle (Saale), Germany; Thomas.Bartels@sportklinik-halle.de; 4 School of Health and Life Sciences, University of the West of Scotland, Glasgow G72 0LH, UK; lawrence.hayes@uws.ac.uk; 5 Department of Sports Science, Martin-Luther-University Halle-Wittenberg, Von-Seckendorff-Platz 2, 06120 Halle (Saale), Germany; bouhafs.elghali@gmail.com; 6 Department of Orthopaedic and Trauma Surgery, Martin-Luther-University Halle-Wittenberg, Ernst-Grube-Str. 40, 06120 Halle (Saale), Germany; rene.schwesig@uk-halle.de

**Keywords:** team sports, muscle power, complex performance diagnostic, training intervention

## Abstract

This study examined the effects of 8 weeks of twice-weekly combined plyometric and sprint with change-of-direction (CPSCoD) training into habitual training regimes of young male soccer players. Participants were randomly allocated to an experimental group (*n* = 17, age: 14.6 ± 0.44 years, body mass: 61.2 ± 7.34 kg, height: 1.67 ± 0.09 m, body fat: 11.2 ± 1.56%) and a control group (*n* = 16, age: 14.6 ± 0.39 years, body mass: 61.1 ± 3.96 kg, height: 1.67 ± 0.05 m, body fat: 11.8 ± 1.47%). Measures obtained pre- and post intervention included vertical and horizontal jump performance (i.e., squat jump (SJ), countermovement jump (CMJ), and standing long jump (SLJ)), and sprint performance (i.e., 5 m and 20 m sprint). In addition, Measures obtained pre- and post-intervention included change-of-direction ability (4 × 5 m sprint test (S 4 × 5 m) and sprint 9–3–6–3–9 m with backward and forward running (SBF)), repeated change of direction (RCoD), and static balance performance (stork balance test). The training group experienced superior jump (all *p* < 0.05; d ≥ 0.61), sprint (all *p* < 0.05; d ≥ 0.58), change-of-direction (CoD) ability (all *p* < 0.05; d ≥ 0.58), RCoD (all parameters except the fatigue index *p* < 0.01; effect size (d) ≥ 0.71), and static balance (all *p* < 0.05; d ≥ 0.66) improvement. Adding twice-weekly CPSCoD training to standard training improves the anaerobic performance of U15 male soccer players.

## 1. Introduction

Fitness is an important determinant of soccer performance [1]. During a 90 min match, elite young soccer players (13–18 years) complete intermittent running and often cover more than 6 km, emphasizing the importance of the aerobic metabolic pathway [2]. In a similar vein, players commonly perform high-speed actions over short distances (i.e., sprinting, acceleration, and deceleration), and their particular movements are associated with soccer-specific actions such as tackling, defending, or creating space during possession, with sprints being the most common action before scoring a goal [3,4,5]. The distance of six locomotor categories (CATs) and the total distance (TD) were chosen as performance results [6]. The following CATs were analyzed: standing (0.0–0.7 km/h), walking (0.7–7.2 km/h), jogging (7.2–14.4 km/h), running (14.4–19.8 km/h), high-speed running (19.8–25.2 km/h), and sprinting (>25.2 km/h). To quantify high-to-moderate accelerations, the number of peaks above a threshold of 2 ms^−2^ was determined [7,8]. In this context, Di Salvo et al. [9] investigated the physical demands of Spanish Premier League football players in relation to their playing position. These researchers reported that wide (11,990 m) and central (12,027 m) midfielders covered the greatest distance. In addition, they reported that wide midfielders covered the greatest distances in high-intensity speed zones (19.1–23.0 km/h: 738 m; >23.0 km/h: 446 m), as well as central midfielders had the highest volume in the mid-intensity zones (11.1 to 14.0 km/h: 1965 m; 14.1 to 19.0 km/h: 2116 m). Furthermore, soccer players complete an average of 50 turns per match [10] and complete a total of 723 ± 203 turns and dodges in a match [11].

Analysis of physical matches has shown that in elite soccer matches, players perform a significant number of high-intensity changes of direction (CoDs) using a broad spectrum of turning angles [11,12]. Some authors have proposed that the number and quality of CoDs in a match influence the outcome of the match in professional soccer [13,14]. Possibly as a result of this association, the ability to perform CoDs in a match is considered an important fitness component in all soccer abilities, and levels of competitions [14,15,16]. Recently, Lloyd et al. [17] reported that the application of optimal CoD training stimulus during the duration of athlete development was crucial for effective programming and improvement of CoD performance in youth. Several studies have reported CoD training improved CoD ability, [11,13]. However, more encouraging for practitioners is the efficacious nature of CoD training on other parameters such as jumping performance, sprint performance, and balance ability in young soccer players [11,13].

Plyometric training is effective at improving jump performance [14] (the mean relative improvement varied between 14% and 29% on vertical jump), given the specific nature of the training method [14,15,16]. However, much similar to CoD, the cross-training effects on CoD ability and sprint performance are encouraging for practitioners and athletes alike [14,15,16]. In addition to performance benefits, plyometric training also exerts healthogenic effects such as increased bone mineral density, improved neuromuscular function, body mass control, improved psychosocial well-being, improved cardiovascular risk profile, and decreased risk of [18,19,20]. A recent systematic review [21] indicated that a twice-weekly plyometric training for 8 to 10 weeks with a 72 h rest period between training sessions improves high-intensity physical abilities (e.g., jump, sprint, agility) in the young soccer player population aged 10 to 17 years, in both sexes.

Despite the increasingly abundant literature on combined training in several sport disciplines and different age categories, there is little research related to applications of this training method in young male soccer players [22,23,24,25]. Recently, Aloui et al. [22] studied the effects of 8 weeks bi-weekly combined plyometric and sprint with change-of-direction (CPSCoD) training in elite under-17 (U17) soccer players. Jumping (Δ19% on a squat jump; Δ21% on countermovement jump), sprinting (Δ−11% on 5 m; Δ−8% on 20 m) and changing-direction ability (Δ−9% sprint 4 × 5 m test), repeated sprinting ability performance (Δ−7% on fastest time; Δ−8% on mean time), and balance performance improved as a result of this training method. Additionally, Beato et al. [23] noted that CPSCoD training improved jump and sprint performance in elite U19 soccer players. Additionally, Jlid et al. [26] reported multidirectional plyometric training improved jump performance, CoD ability, and dynamic postural control in prepubertal soccer players.

Despite the efficacious nature of CPSCoD training to enhance anaerobic fitness in soccer, there are only a handful of studies that have examined this phenomenon, and mostly in adolescents >15 years of age. Therefore, the purpose of this investigation was to examine the effects of 8 weeks of twice-weekly CPSCoD training on anaerobic performance (i.e., jumping, speed, and CoD ability) and balance ability in elite male U15 soccer players during the competitive season. Particular tests of interest were vertical and horizontal jump performance (i.e., squat jump (SJ), countermovement-jump (CMJA), standing long jump test (SLJ)), sprint performance (i.e., 5 m and 20 m sprint), and CoD ability (4 × 5 m sprint test (S 4 × 5 m) and sprint 9–3–6–3–9 m with backward and forward running (SBF). In addition, repeated CoD (RCoD) ability and static balance performance (stork balance test) were examined. We hypothesized a priori that CPSCoD would improve physical fitness, particularly jump performance, sprint performance, and CoD ability, when compared with a control group, who maintained regular training during the season.

## 2. Materials and Methods

### 2.1. Participants

The 33 participants of all playing positions from a single male soccer team in the first national division took part in this study. Participants had 4.6 ± 0.9 years of systematic soccer training background comprising of five training sessions per week. Participants were examined by the team physician to ensure players were healthy enough to complete CPSCoD training. Participants were randomly assigned between an experimental group (*n* = 17, age: 14.6 ± 0.44 years, body mass: 61.2 ± 7.3 kg, height: 1.67 ± 0.09 m, body fat: 11.2 ± 1.6%) and a control group (*n* = 16, age: 14.6 ± 0.4 years, body mass: 61.1 ± 4.0 kg, height: 1.67 ± 0.05 m, body fat: 11.8 ± 1.5%). Participants were free from injury during the 6-month period before the beginning of the study and throughout the study. No baseline inter-group differences existed for anthropometric characteristics between groups (*p* ≥ 0.05).

Prior to the study, participants had completed a 6-week preparation period (pre-season training) (5–6 sessions per week). The first 3 of these weeks were to focus on improving aerobic capacity by following low-to-moderate intensity interval training, and on improving resistance and muscle strength by following low-to-moderate intensity circuit training resistance. The last 3 weeks have been focused on improving aerobic power by following high-intensity interval training (HIIT) and small-sided games, and improving muscle power by following high-intensity circuit training, supplemented participation in a non-competitive training match.

Procedures were approved by the University Institutional Review Committee for ethical human experimentation (reference number: KS000002020 and date of approval 10 December 2020). Participants (and their guardians, in the case of minors) provided informed written consent. Two familiarization sessions were conducted 2 weeks before testing, which was 2 months into the competitive season.

### 2.2. Experimental Design

Participants’ only physical training during the experimental phase was associated with the soccer team and one weekly 60 min school physical education session. The usual micro-training cycle for both groups consists of five sessions per week (approximately 90 min each session), with a competitive game each weekend. During the first three sessions of micro-cycle training each week, approximately 60% of the total time of the session was focused on technical–tactical training, as well as 40% of the time was focused on physical training. In addition, during the last two training sessions of each week, approximately 75% of the micro-cycle of training was focused on technical–tactical training, as well as 25% of the time was focused on physical training (Table 1).

The control group maintained its normal training program throughout the intervention of 8 weeks. While every Tuesday and Thursday, the experimental group replaced the technical–tactical part of their standard training program with a combined plyometric and sprint with CoD training. Indeed, the first session of the micro-cycle training of each week was focused on the development of aerobic capacity through skill drills/circuit training with medium volume and low intensity. The second training session included HIIT and small-sided games aimed at developing maximum aerobic power [27]. The third training session included dynamic exercises based on bodyweight only and jumping and sprinting exercises aimed at maximum anaerobic power. The fourth and fifth training sessions of each week included agility and vivacity training, respectively.

The investigation was completed during a local soccer season in 2020/2021.

### 2.3. Details of Combined Plyometric and Short Sprints with Change-of-Direction Training

**Figure 1:**
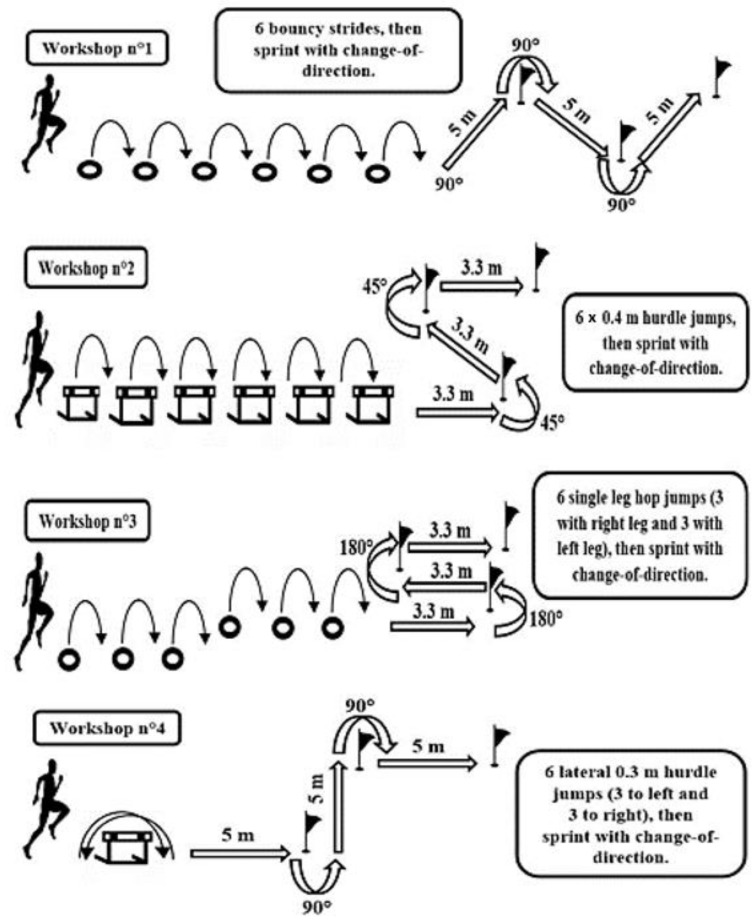
Exercise used in combined plyometric and sprint with a change-of-direction training program.

The CPSCoD training program consisted of four drills, with increasing repetitions over the training period, twice per week (as visualized in Figure 1 and quantified in Table 2).

Each CPSCoD drill began with plyometric exercises and finished with a sprint with CoD. It should be noted that participants were used to applying plyometrics and CoD drills and had achieved good technical competency through training activities before starting the investigation.

Plyometric training variables were based on recommendations for volume and intensity from Bedoya et al. [21]. Furthermore, CoD angles were based on previously published recommendations [23].

### 2.4. Testing Schedule

Tests were conducted at least 3 days after the last competitive match and 5–9 days after the previous training session. Testing was conducted on a tartan surface, which was part of the players’ weekly training schedule. Standardized warm-up preceded each test. Tests were completed over three separate testing days in the following order: anthropometric assessment, squat jump (SJ), countermovement jump (CMJ), sprint 4 × 5 m test (S 4 × 5 m) (all day 1); the stork balance test, 5 and 20 m sprint, and sprint 9–3–6–3–9 m with backward and forward running (SBF) (all day 2); the standing long jump (SLJ) and the repeated CoD ability (RCoD) (both day 3).

The 5 and 20 m sprint performance, S 4 × 5 m, SBF, SJ, CMJ, SLJ, stork balance test, and the RCoD have all been previously described in detail [28,29,30] and therefore are not detailed here to avoid self-plagiarism. Anthropometric characteristics (body mass and body fat percentage) were evaluated after an overnight fast, in the morning (7–8 am), bare foot, with the bioelectrical impedance analysis (BIA) method (BC-602, Tanita Co., Tokyo, Japan) [31].

### 2.5. Statistical Analyses

All analysis was conducted on SPSS version 25.0 for Windows (IBM, Armonk, NY, USA). Normal distribution was tested using the Shapiro–Wilk test. Homogeneity of variance was determined using Levene’s test. Independent samples *t*-tests examined between-group differences at baseline. An effect of training was considered by a mixed method of two-way (time × group) analysis of variance (ANOVA) with repeated measures. Subsequently, dependent samples *t*-tests tested for within-group training changes from pre- to post intervention were examined with Tukey’s post hoc procedure applied to avoid type I error. Alpha level is reported as exact *p* values, as suggested by the American Statistical Association [31]. The effect size for paired comparisons is reported as Cohen’s d [32] and interpreted as trivial (<0.35), small (≥0.35–0.80), moderate (≥0.80–1.50), and large (≥1.50), based on the recommendations of Rhea [33] for recreationally trained subjects. Percentage changes were calculated as ((post-training value−pre-training value)/pre-training value) ×100. Reliability was evaluated using intraclass correlation coefficients (ICC) [34] and the coefficients of variation (CV) over consecutive pairs of intra-participant trials [35]. All performance measures had an ICC > 0.80 and a CV < 5% (Table 3). Data are reported as mean ± standard deviation (SD).

## 3. Results

### 3.1. Normal Distribution and Homogeneity of Variance

The results of the Shapiro–Wilk exhibited approximately half the parameters (14/29, 48%) were not normally distributed. The following parameters were not normally distributed: height (*p* = 0.002), body fat (*p* = 0.030), SJ 1 (*p* = 0.036), SJ 2 (*p* = 0.001), SLJ 2: *p* = 0.014, sprint 20 m 2 (*p* = 0.033), RSA_best_ 2 (*p* = 0.011), RSA_mean_ 2 (*p* = 0.020), RSA fatigue index 1 (*p* = 0.037), RSA fatigue index 2 (*p* = 0.003), and all stork balance parameters (*p* < 0.001).

In total, 33% (4/12) of variance tests indicated heterogeneity of variance (SJ: *p* = 0.003, CMJ: *p* = 0.048, RSA Fatigue index: *p* = 0.005, and stork balance of left leg: *p* = 0.040).

### 3.2. Reliability

Measured reliability is displayed in Table 3. There were no baseline between-group differences.

### 3.3. Effect of Training on Jump Performance

In terms of vertical and horizontal jump performance, the present results showed significant intervention effects (group × time interaction), in the experimental group compared to the control, (Δ22%; *p* < 0.05; d = 0.61 on squat jump Δ20%; *p* < 0.05; d = 0.71 on countermovement jump and Δ15%; *p* < 0.05; d = 0.66 on standing long jump test, respectively, for the experimental group) (Table 4). 

### 3.4. Effect of Training on Sprint Performance

There was a group × time interaction in sprint performance with the experimental group (EG) improving more than the control group (CG) over distances of 5 (Δ−3%; *p* < 0.001; d = 0.39 and Δ−11%; *p* < 0.001; d = 1.81) and 20 m (Δ−3%; *p* < 0.001; d = 0.48 and Δ−10%; *p* < 0.001; d = 1.51), respectively, for the EG (Table 4).

### 3.5. Effect of Training on Change-of-Direction Ability

The sprint 4 × 5 m test displayed the largest group × time interaction effect (*p* = 0.002, d = 0.82) for all jump and change-of-direction performance parameters (Table 4). Especially, the EG (*p* < 0.001, d = 2.12) benefited from the intervention compared with the CG (*p* < 0.001, d = 0.68).

The SBF test showed an intervention effect with the EG improving more than CG (Δ−3%; *p* < 0.001; d = 0.62 and Δ−8%; *p* < 0.001; d = 1.65, respectively, for the EG) (Table 4).

### 3.6. Effect of Training on Repeated Change-of-Direction Ability

The RCoD test showed group × time interactions for RCoD_fastest_ (*p* = 0.006, d = 0.72) and RCoD_mean_ (*p* = 0.006, d = 0.71). Over the time, the improvement of performance was much higher in the EG (d_fastest_ = 2.01, d_mean_ = 2.02) than in the CG (d_fastest_ = 0.42, d_mean_ = 0.43) (Table 5).

### 3.7. Effect of Training on Balance Performance

Balance performance was improved more in the experimental group, compared with the control group (right leg: Δ68%; *p* < 0.05; d = 0.66; left leg: Δ79%; *p* < 0.01; d = 0.72, respectively for the experimental group) (Table 5).

## 4. Discussion

This investigation examined the effect of 8 weeks of CPSCoD training on anaerobic performance and balance ability in elite U15 soccer players. We observed that adding CPSCoD to habitual training enhanced jump performance, sprint performance, CoD ability, RCoD ability, and balance performance.

### 4.1. Effect of Training on Jump Performance

In soccer, vertical jumps are performed when a player jumps toward a ball in an attempt to head direct the ball for an attacking (scoring/passing) or defensive (clearance/interception) task and is decisive action during matches [32]. We observed intervention effects in the experimental group, compared with the control, on vertical and horizontal jump performance (Table 4). The positive effects of CPSCoD in the present investigation are in line with Aloui et al. [22], who studied the effects of 8 weeks of CPSCoD training on U17 male soccer players and found this type of training improved vertical and horizontal jump performance. In addition, Beato et al. [23] reported significant increases in horizontal jump performance following CPSCoD training, in male young soccer players (U18). Although mechanisms for adaptation cannot be fully determined in the present study, it is likely improved jump performance was mediated by neural adaptation such as motor unit recruitment and the Hoffman reflex [20,33]. We suggest eccentric muscular contractions necessitated by the plyometric training are able to produce greater force and thus may provide a large stimulus above and beyond concentric contractions alone [34].

### 4.2. Effect of Training on Sprint Performance

In professional soccer match play, sprints in a straight line (45%), followed by vertical jumping (16%) were the two most commonly observed actions immediately prior to a goal being scored [35]. It is well known that muscle strength and contraction speed (i.e., muscle power) are determining factors in sprint speed [36,37,38], and the improved sprint ability evoked by CPSCoD training in the present study may translate to enhanced match play abilities and preferential match outcomes. We observed intervention effects in the EG, compared with the control for CoD ability (Table 4). Our results are aligned with those of Hammami et al. [28], who reported improved sprint performance over 5 and 20 m following CPSCoD training in young male handball players. Similarly, Michailidis et al. [25] observed improved 10 m, but not 30 m, sprint performance after CPSCoD training in prepubertal male soccer players. Improvement in sprinting performance after CoD training could be due to improved leg extensor power and the ability to produce lower limb strength more effectively after training [39,40]. In addition, temporal sequencing of muscle activation for more efficient movement, preferential recruitment of the fastest motor units, or increased nerve conduction speed promoted by plyometric training may be responsible for the improved sprint times [41].

### 4.3. Effect of Training on Change-of-Direction Ability

The CoD improvement observed herein following CPSCoD training is encouraging for trainers, as CoD ability is an important trait in competitive soccer [42,43]. Our data, especially the moderate interaction (d = 0.82) and the large (d = 2.12) effect for the EG regarding sprint 4 × 5 m, are in accordance with the existing literature, which has demonstrated improved CoD ability after several programs, including CPSCoD training [22,23,24], plyometric training [28,44], and CoD training [45,46]. Recently, Michailidis et al. [25] and Makhlouf et al. [24] reported improved CoD ability following CPSCoD training in prepubertal males. Improvements may have been caused by reduced contact times as a result of increased muscle strength and power, and efficiency of movement, as this has been noted previously following plyometric [20,47]. Similarly, enhanced eccentric lower limb strength could enhance the ability to accelerate and decelerate during CoDs [48]. In this context, Chaalali et al. [45] suggested the observed CoD enhancement may be related to improved CoD technique and increased strength and power of the leg extensors, after CoD training.

### 4.4. Effect of Training on Repeated Change-of-Direction Ability

Soccer players must complete high-intensity repeated efforts and/or sprints to compete at a high level [49]. We reported improved RCoD ability in the EG, compared with the CG for all parameters except the fatigue index. Reinforcing our results, Aloui et al. [22] observed improvement in RCoD ability following CPSCoD training in male U17 soccer players. Conversely, Hammami et al. [28] reported no improvement in RCoD ability following CPSCoD training in U15 male handball players. Improved RCoD observed herein was likely resultant from improved anaerobic fitness such as CoD ability, sprint ability, and jumping ability [50,51]. Particularly, RCoD ability improvements were most likely the result of improvements in stretch-shortening cycle efficiency, motor unit synchronization, or tendon stiffness [52].

### 4.5. Effect of Training on Balance Performance

Balance is the ability to maintain postural control during an and is classified can be classified as dynamic and static [53]. In soccer, players benefit from the ability to regain postural control to attenuate injury risk while landing from a dynamic movement, such as a pronounced deceleration, cut, or jump [54]. The EG in this study improved the static balance, compared with the control group, which confirms the results of Aloui et al. [22], who observed improved static balance after CPSCoD training in U17 male soccer players. Recently, Makhlouf et al. [18] reported improved static and dynamic balance following CPSCoD training in prepubertal male soccer players. These adaptations may be caused by neural factors such as improved reaction time or precision of movement [55]. Another possible reason for improved balance performance could be the increase in muscle strength of the lower limbs, the increase in muscle coordination, and the facilitation of fast-twitch motor units [56]. Ondra et al. [57] reported that a high level of postural stability is believed to be the factor reducing the risk of lower limb injury, emphasizing the importance of balance in this cohort.

### 4.6. Limitations

Results of this study suggest CPSCoD training confers advantages in anaerobic capacity and balance performance in elite U15 soccer players. These mostly small adaptations were observed after only 8 weeks, alongside other in-season training, and should be related to the higher risk of injury from performing plyometric movements. Thus, CPSCoD training during the in-season period may promote reduced injury risk in addition to improved anaerobic fitness. However, before we can promote CPSCoD training for all soccer squads, a larger confirmatory study may be required to elucidate if these effects are manifest across all age groups, players levels, and sexes. Moreover, as our participants were already well-trained and well-versed in the type of training included in the CPSCoD program, caution should be exercised when inducting untrained or novice athletes onto a CPSCoD training program.

## 5. Conclusions

CPSCoD training enhanced balance performance and anaerobic capacity (jumping, sprinting, CoD ability, and RCoD ability) during the competition season. However, 83% (10/12) of the interaction effects were small. Only for sprint 4 × 5 m, a moderate effect was observed. Note that during the intervention period, no injury was reported. Therefore, CPSCoD training can be considered safe for well-trained and well-versed young male soccer players provided progression guidelines are followed.

## Figures and Tables

**Table 1:** Details of general training routine during the week performed by both control and experimental groups over the 8-week intervention.

Days	Objectives
Mondays	Rest
Tuesdays	Aerobic capacity training and defensive tactics training
Wednesdays	Aerobic power training and defensive tactics training
Thursdays	Power anaerobic training and defensive and offensive tactics training
Fridays	Agility training and technical training and offensive tactics training
Saturdays	Vivacity training and technical training and offensive tactics training
Sunday	Official matches

**Table 2:** Plyometric components were introduced into the program of the experimental group.

Week	Drill 1	Drill 2	Drill 3	Drill 4	Total (Contact)
1	3 Repetitions	3 Repetitions	3 Repetitions	3 Repetitions	72
2	3 Repetitions	3 Repetitions	3 Repetitions	3 Repetitions	72
3	4 Repetitions	4 Repetitions	4 Repetitions	4 Repetitions	96
4	4 Repetitions	4 Repetitions	4 Repetitions	4 Repetitions	96
5	5 Repetitions	5 Repetitions	5 Repetitions	5 Repetitions	120
6	5 Repetitions	5 Repetitions	5 Repetitions	5 Repetitions	120
7	6 Repetitions	6 Repetitions	6 Repetitions	6 Repetitions	144
8	6 Repetitions	6 Repetitions	6 Repetitions	6 Repetitions	144

All repetitions and sessions were separated by 90 s recovery intervals.

**Table 3:** Interclass correlation coefficient (ICC, 95% confidence intervals (95% CI)) and coefficient of variation (CV) showing acceptable reliability for all performance. ICC > 0.75 and CV < 10% marked in bold.

	ICC (95% CI)	CV (95%CI) [%]
*Sprint times*		
5 m (s)	**0.93** (0.90–0.97)	**2.0** (1.7–2.4)
20 m (s)	**0.94** (0.91–0.97)	**1.9** (1.5–2.3)
*Change of direction*		
Sprint 4 × 5 m (s)	**0.89** (0.84–0.93)	**2.2** (1.8–2.7)
SBF (s)	**0.87** (0.83–0.91)	**2.1** (1.7–2.5)
*Vertical jump*		
SJ (cm)	**0.95** (0.90–0.98)	**2.4** (1.9–2.8)
CMJ (cm)	**0.94** (0.89–0.98)	**2.8** (2.4–3.2)
*Horizontal jump*		
SLJ (m)	**0.91** (0.87–0.95)	**3.8** (3.4–4.3)
*Stork balance test*		
Right leg (s)	**0.82** (0.72–0.88)	**4.7** (4.3–5.4)
Left leg (s)	**0.80** (0.70–0.87)	**4.9** (4.4–5.5)

ICC: Intraclass Correlation Coefficients; 95% CI: 95% Confidence Intervals; CV: Coefficients of Variation; SBF: Sprint 9–3–6–3–9 m with backward and forward running; SJ: Squat Jump; SLJ: Standing Long Jump.

**Table 4:** Vertical and horizontal jump tests, sprint times, and change-of-direction performance performances in experimental and control groups before and after the 8-week intervention.

	Experimental (*n* = 17)			Paired *t*-Test		Control (*n* = 16)			Paired *t*-Test		ANOVA (Group × Time)	
	Pre	Post	% Δ	*p*	ES	Pre	Post	% Δ	*p*	ES	*p*	ES
Vertical jump												
SJ (cm)	25.1 ± 4.00	30.1 ± 4.75	20.2 ± 1.22	<0.001	1.15	24.5 ± 2.06	25.4 ± 2.16	3.70 ± 1.21	<0.001	0.43	0.018	0.61 (small)
CMJ (cm)	27.7 ± 3.66	33.2 ± 3.90	20.1 ± 2.26	<0.001	1.46	27.5 ± 2.50	28.6 ± 2.55	3.94 ± 1.42	<0.001	0.43	0.007	0.71 (small)
Horizontal jump												
SLJ (m)	1.81 ± 0.13	2.08 ± 0.11	14.8 ± 2.78	<0.001	2.28	1.78 ± 0.18	1.85 ± 0.17	4.31 ± 1.68	<0.001	0.43	0.012	0.66 (small)
Sprint												
5 m (s)	1.28 ± 0.10	1.12 ± 0.06	−11.3 ± 2.35	<0.001	1.81	1.24 ± 0.09	1.21 ± 0.09	−2.82 ± 0.71	<0.001	0.39	0.013	0.65 (small)
20 m (s)	3.61 ± 0.26	3.26 ± 0.19	−9.52 ± 1.30	<0.001	1.51	3.59 ± 0.21	3.49 ± 0.21	−2.81 ± 0.61	<0.001	0.48	0.027	0.58 (small)
Change of direction Performance												
Sprint 4 × 5 m (s)	6.60 ± 0.32	5.97 ± 0.27	−9.39 ± 0.83	<0.001	2.12	6.60 ± 0.26	6.42 ± 0.27	−2.71 ± 0.72	<0.001	0.68	0.002	0.82 (moderate)
SBF (s)	9.09 ± 0.46	8.34 ± 0.44	−8.19 ± 0.78	<0.001	1.65	9.09 ± 0.42	8.83 ± 0.41	−2.68 ± 0.68	<0.001	0.62	0.026	0.58 (small)

ES: effect size; ANOVA: Analyze of Variance; CMJ: Countermovement Jump; Δ: percentage difference; *t*-test: *t* is the calculated difference in units of the standard error.

**Table 5:** Repeated change-of-direction ability and stork balance test performances in experimental and control groups before and after the 8-week intervention.

	Experimental (*n* = 17)			Paired *t*-Test		Control (*n* = 16)			Paired *t*-Test		ANOVA (Group × Time)	
	Pre	Post	% Δ	*p*	ES	Pre	Post	% Δ	*p*	ES	*p*	ES
Repeated Change of Direction Ability parameters												
Fastest time (s)	7.14 ± 0.29	6.57 ± 0.40	−7.85 ± 0.67	<0.001	2.01	7.15 ± 0.31	7.01 ± 0.33	−1.93 ± 0.56	<0.001	0.42	0.006	0.72 (small)
Mean time (s)	7.28 ± 0.31	6.69 ± 0.41	−7.85 ± 0.65	<0.001	2.02	7.26 ± 0.32	7.12 ± 0.34	−1.94 ± 0.58	<0.001	0.43	0.006	0.71 (small)
Fatigue index (%)	1.97 ± 0.79	1.93 ± 0.40	1.96 ± 13.8	0.408	0.10	1.51 ± 0.32	1.49 ± 0.24	0.47 ± 9.91	0.844	0.04	0.844	0.06 (trivial)
Stork balance test												
Right leg (s)	11.9 ± 4.39	19.6 ± 6.12	67.6 ± 10.8	<0.001	1.45	12.9 ± 4.04	14.6 ± 4.06	14.4 ± 7.07	<0.001	0.42	0.012	0.66 (small)
Left leg (s)	10.4 ± 4.28	18.2 ± 6.00	78.6 ± 14.3	<0.001	1.50	10.5 ± 3.03	12.3 ± 3.17	18.0 ± 8.11	<0.001	0.58	0.006	0.72 (small)

## Data Availability

The raw data supporting the conclusions of this article will be made available by the authors without undue reservation.
